# Performance Limitations of Relay Neurons

**DOI:** 10.1371/journal.pcbi.1002626

**Published:** 2012-08-09

**Authors:** Rahul Agarwal, Sridevi V. Sarma

**Affiliations:** Department of Biomedical Engineering, Johns Hopkins University, Baltimore, Maryland, United States of America; University of Freiburg, Germany

## Abstract

Relay cells are prevalent throughout sensory systems and receive two types of inputs: driving and modulating. The driving input contains receptive field properties that must be transmitted while the modulating input alters the specifics of transmission. For example, the visual thalamus contains relay neurons that receive driving inputs from the retina that encode a visual image, and modulating inputs from reticular activating system and layer 6 of visual cortex that control what aspects of the image will be relayed back to visual cortex for perception. What gets relayed depends on several factors such as attentional demands and a subject's goals. In this paper, we analyze a biophysical based model of a relay cell and use systems theoretic tools to construct analytic bounds on how well the cell transmits a driving input as a function of the neuron's electrophysiological properties, the modulating input, and the driving signal parameters. We assume that the modulating input belongs to a class of sinusoidal signals and that the driving input is an irregular train of pulses with inter-pulse intervals obeying an exponential distribution. Our analysis applies to any 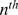 order model as long as the neuron does not spike without a driving input pulse and exhibits a refractory period. Our bounds on relay reliability contain performance obtained through simulation of a second and third order model, and suggest, for instance, that if the frequency of the modulating input increases or the DC offset decreases, then relay increases. Our analysis also shows, for the first time, how the biophysical properties of the neuron (e.g. ion channel dynamics) define the oscillatory patterns needed in the modulating input for appropriately timed relay of sensory information. In our discussion, we describe how our bounds predict experimentally observed neural activity in the basal ganglia in (i) health, (ii) in Parkinson's disease (PD), and (iii) in PD during therapeutic deep brain stimulation. Our bounds also predict different rhythms that emerge in the lateral geniculate nucleus in the thalamus during different attentional states.

## Author Summary

In cellular biology, it is important to characterize the electrophysiological dynamics of a cell as a function of the cell type and its inputs. Typically, these dynamics are modeled as a set of parametric nonlinear ordinary differential equations which are not always easy to analyze. Previous studies performed phase-plane analysis and/or simulations to understand how constant inputs impact a cell's output for a given cell type. In this paper, we use systems theoretic tools to compute analytic bounds of how well a single neuron's output relays a driving input signal as a function of the neuron type, modulating input signal, and driving signal parameters. The methods used here are generally applicable to understanding cell behavior under various conditions and enables rigorous analysis of electrophysiological changes that occur in health and in disease.

## Introduction

**Figure 1:**
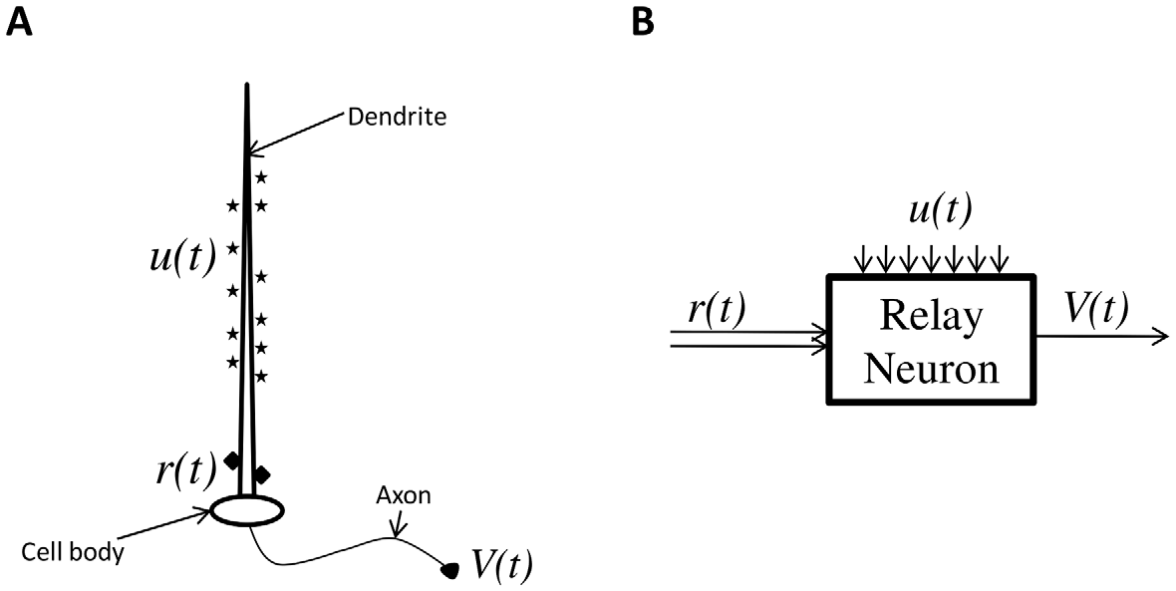
A relay neuron. (**A**) Illustrating a relay neuron. Ensemble activity of all the distal synapses (stars) is modulating input 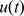. The proximal synapses (diamonds) form the driving input 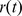. The output is the axonal voltage 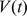. (**B**) A block diagram of a relay neuron showing two inputs and output 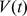.

Relay neurons are found in various brain nuclei including the thalamus [1]–[3]. Experiments have suggested that the inputs to a thalamic relay neuron can be divided into two categories: driving and modulating. The driving input typically contains sensory information (e.g visual, motor) and the modulating input controls relay of this sensory information back to cortex [4]. The driving input is made up of a few synapses on the proximal dendrites whereas the modulating input comprises all other synapses [5], [6] (see Figure 1 A).

For example, the lateral geniculate nucleus (LGN) in the thalamus receives the driving input from the retina and projects to the primary visual cortex. The modulating input comprises descending inputs from layer 6 of the visual cortex and ascending inputs from the brain stem. The function of the LGN is to selectively relay sensory information from the retina subject to attentional needs [4], [7]. It has been observed that during different attentional needs (which translate into different relay demands), local field potentials (LFPs) in the LGN have a concentration of power in different frequency bands (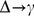) [8], [9]. LFPs may be reflected in the modulating input because they are believed to arise from ensemble synaptic activity [10]. This would then suggest that one mechanism that controls relay in the LGN cell is the frequency of the modulating input.

Similarly, relay neurons in the motor thalamus receive driving inputs from sensorimotor cortex, and modulating inputs from the basal ganglia (BG), specifically the Globus Pallidus internal segment (GPi) [4], [11]. The driving input contains information about the actual movement via proprioception, and the modulating input facilitates/impedes relay of this information to motor cortex [12]–[16]. It has been observed that neural activity in the GPi changes its oscillatory patterns when a subject's cognitive state moves from being idle to planning a movement [17]. In particular, GPi activity has prominent beta band oscillations when the subject is idle, which then get suppressed when the subject plans to move. This suppression coincides with an emergence of gamma band oscillations. This would suggest, again, that one mechanism that controls relay in the motor thalamic cell is the frequency of the modulating input.

In this study, we set out to quantify when and how these thalamic cells relay driving inputs. Previous attempts to study relay neurons are made in [15], [16], [18]–[20]. Specifically, in [18], [19] in-vitro experiments are used to understand how background synaptic input modulates relay reliability of a thalamic neuron. These studies suggest that the neuron's reliability of relaying an incoming spike is governed by the background synaptic input (the modulating input) combined with intrinsic properties of the neuron. In particular [19], showed that if the variance of the background synaptic input increases, the transmission reliability goes down, and [18] showed that the feedback inhibition from the nucleus reticularis modulates the excitability of the thalamic cell membrane and hence gates transmission of spikes from the retina.

An attempt to analytically study relay neurons is made in [15], where in they studied the effects of BG inhibition on the thalamic relay reliability. They used a 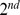 order non-bursting model and phase-plane analysis to study relay neuron properties. However, they only considered a constant and a low frequency periodic modulating input. Additionally, only one deterministic periodic waveform was considered for the driving input. A follow up study with a similar objective is presented in [20], wherein the authors analyzed a relay neuron driven only by a driving input (no modulating input). Using Markov models, they studied how different distributions of driving pulse arrival times affect relay reliability. However, they did not present an explicit expression for the dependence of reliability upon input distributions and relay neuron properties.

The work presented here is different from the above computational studies in that we include classes of modulating and driving inputs in our analysis, and we employ systems theoretic tools to obtain explicit analytical bounds on reliability as a function of the neuron's electrophysiological properties (i.e., model parameters), the modulating input signal, and the driving signal parameters. Our analysis is applicable to any 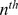 order model as long as the neuron does not spike without a pulse in the driving input and exhibits a refractory period. Consequently, our analysis is relevant for relay cells whose electrophysiological dynamics, including bursting, may be governed by several different ion channels and is more rigorous than previous works. Our lower and upper bounds contained reliability computed through simulation of both a second- and third-order model, and suggest, for example, that if the frequency of the modulating input increases and/or its DC offset decreases, then relay reliability increases.

The methods used here are generally applicable to understanding cell behavior under various conditions. In the discussion section, we show how our analysis shed new insights into motor signal processing in health and in Parkinson's disease with and without therapeutic deep brain stimulation. We also discuss how our bounds predict neural activity generated in the LGN during visual tasks with different attentional needs as well as during sleep. In particular, we show how our bounds predict the following observations in the LGN: (i) prominent 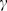 and 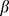 rhythms (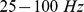) in the LGN LFPs during high attentional tasks [9]; (ii) phase locking between 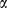 rhythm (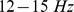) in LFPs and spiking activity in the LGN in awake behaving cats [21]; (iii) 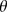 rhythms (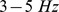) in drowsy cats; and, (iv) even slower 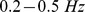 rhythms in sleeping cats [8].

## Materials and Methods

In this section, first we describe a biophysical model of a relay neuron, and then use systems theoretical tools to compute bounds on relay reliability.

### A Relay Neuron Model

A relay neuron receives two kinds of inputs: a driving input, 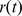 and a modulating input 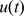, and generates one output, 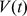, as shown in Figure 1 B. The function of this type of neuron is to generate an output that relays the driving input at appropriate times. The modulating input does as its name implies i.e. it modulates the neuron's ability to relay the driving input [4]. This relay neuron model structure has been widely used to model thalamic relay neurons [15], [16], [22]–[26].

We would like to understand exactly how the modulating input affects relay reliability of the neuron. To do so, we use a biophysical-based model to describe the electro-physiological dynamics of the relay neuron. We first begin with a second order model to highlight structure in the model dynamics, and then we present an 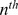 order generalization. Recall that the output of the cell, 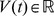, is the membrane voltage of the neuron. Then for time 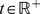,
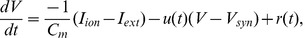
(1a)

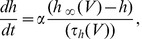
(1b)

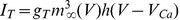
(1c)

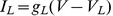
(1d)

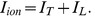
(1e)


In (1), 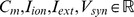 are the membrane capacitance, ionic current, external current and synaptic reversal potential, respectively. 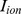 is composed of currents 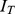, which is a low threshold calcium ion current, and 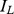 which is the neuron's membrane leakage current. 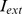 is a constant external current, and 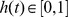, is an internal state of the system representing the probability that a calcium channel inactivation gate is open at a time 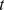. 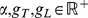 are temperature correction factor, maximum calcium current and leakage current conductance, respectively. The details of 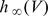, 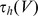 and 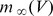 and numerical values used in our simulations are given in Tables 1 and 2. This is a simplified model of a thalamic neuron that is driven only by calcium ion and leak currents. We begin with this model because it is simple and still contains low threshold calcium currents which are shown to govern input selectivity of relay neurons, in a computational study [23]. This model has also been used to model neurons in the inferior olive for the purpose of studying sub-threshold oscillations [27].

**Table 1:** Details of function in (1).

Function	Value
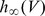	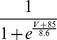
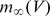	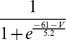
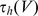	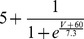

**Table 2:** Parameter's values in (1).

Parameter	Value
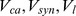	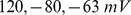
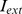	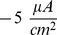
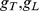	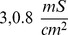
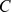	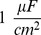

#### State space representation and 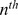 order generalization

By defining a state vector 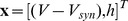, an equivalent state space representation to (1) can be written as:
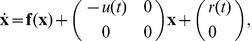
(2) where 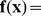


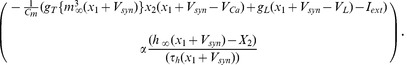
(3)


Note that 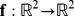 is a non linear, continuous and differentiable vector-valued function of 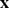. In general, a state space representation takes the form 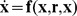, however, there is more structure in (2). From (2), one can see that 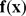 is only a function of the system's internal states. The modulating input, 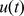, multiplies the first component of the state 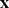, while the driving input, 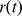, is an exogenous input to the system.

The 2nd order model (2) can be generalized to an 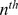 order model to include more ion channels as well as more complicated spiking dynamics such as bursting. The 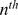 order model is as follows:
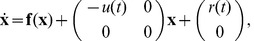
(4)


Here, 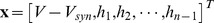 is the n-dimensional state vector of the system, where 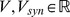 are the membrane and the synaptic reversal potential of the cell, respectively. Each 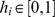, denotes the probability that a 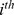 ion gate is open. 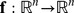 is a nonlinear, continuous and differentiable vector-valued function of 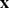 with following form:
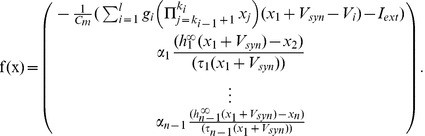
(5)


Each 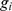 is the conductance of the 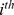 ion channel. 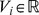 is the reversal potential of 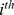 ion. 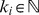 are such that 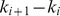 are the number of gates in the 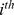 ion channel and 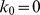. Each 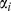 is a temperature correction factor. 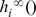 and 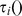 are functions similar to 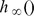 and 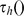.

#### Inputs and outputs

For our relay reliability analysis, we assume that the two inputs belong to the following classes of signals:

- **Driving Input** 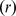: This input represents the spiking activity from other neurons (e.g. cortical neurons), which the neuron must relay. Synapses of the driving input occur on proximal dendrites and are excitatory in nature. The driving input synapses are fewer in number than modulating input synapses. However, the magnitude of post synaptic potential of each driving synapse is larger compared to a modulating input synapse [4], [6]. Therefore, we assume driving input belongs to the following class of functions:
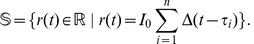
(6) Here, 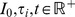 and 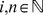. 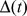 is a Dirac delta function [28]. The 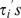 are generated randomly such that 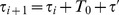, where 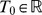 is a constant that represents the refractory period of driving input, and 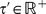 is exponentially distributed with probability density function:
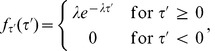
(7) where 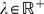. The average inter-pulse interval is 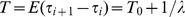. Note that 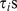 are characterized completely by 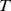 and 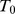. A sample driving input is shown in Figure S1 A (supplementary material).
- **Modulating Input** 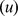: This input modulates the dynamics of the neuron and governs relay performance. Synapses of the modulating input are generally inhibitory and occur on distal dendrites. The magnitude of post synaptic potential of each synapse is smaller as compared to a driving synapse [4], [6]. Therefore, this input is represented in the biophysical model (1) as a synaptic input and belongs to the following class of sinusoidal functions:

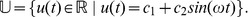
(8) Here 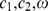, and 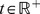 and 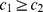. Since 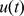 represents a conductance, we impose the constraint 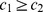 to ensure that 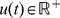. Also, 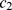 is appropriately small so that the modulating input does not make the relay neuron spike without a driving input pulse. This property of the modulating input will be useful when we linearize (1) for the analysis.
- We model the modulating input in a deterministic manner as it represents the ensemble sum of inhibitory post synaptic potentials (IPSPs). These IPSPs are generally small because inhibitory synapses activate the T-type 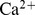 channels allowing an influx of 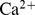 thereby reducing the magnitude of IPSPs at the soma. In relay cells, T-type 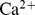 channels have a higher density on distal dendrites [29], and this reduces the magnitude of the IPSPs even further. An ensemble effect [30] of these small IPSPs give rise to a deterministic 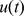. Note that excitatory postsynaptic potentials of driving input will not get attenuated by the T-type 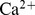 channels as these channels get activated only when the cell is hyperpolarized.
- We choose the class of sinusoidal signals to shed insights into the mechanisms of oscillatory behavior or rhythms of LFPs which are often analyzed in experiments [8], [9], [21]. Note that LFPs arise from ensemble synaptic activity and hence may represent the modulating input. [10]. A sample modulating input is shown in Figure S1 B (supplementary material).
- **Output**: The output of the relay neuron is its membrane voltage 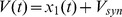.

#### Properties of 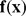


The function 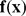 is assumed to have the following 3 properties but is otherwise general:

1. **Stable neuron**: Consider the following undriven system:
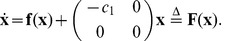
(9) This system is the same as (4) where 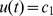 and 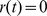. Although, this system is nonlinear, we can study it via linearization about trajectories and/or an equilibrium point.

In general, a non-linear system may have multiple equilibria with different stability properties. But for our purposes, we choose 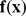 such that (9) has only one globally stable equilibrium point, 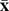, for all pragmatic 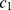. Such a neuron is called a **stable neuron** [27]. This condition ensures that the neuron does not have any limit cycle, therefore, the neuron does not spike without a pulse in 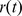.

**Figure 2:**
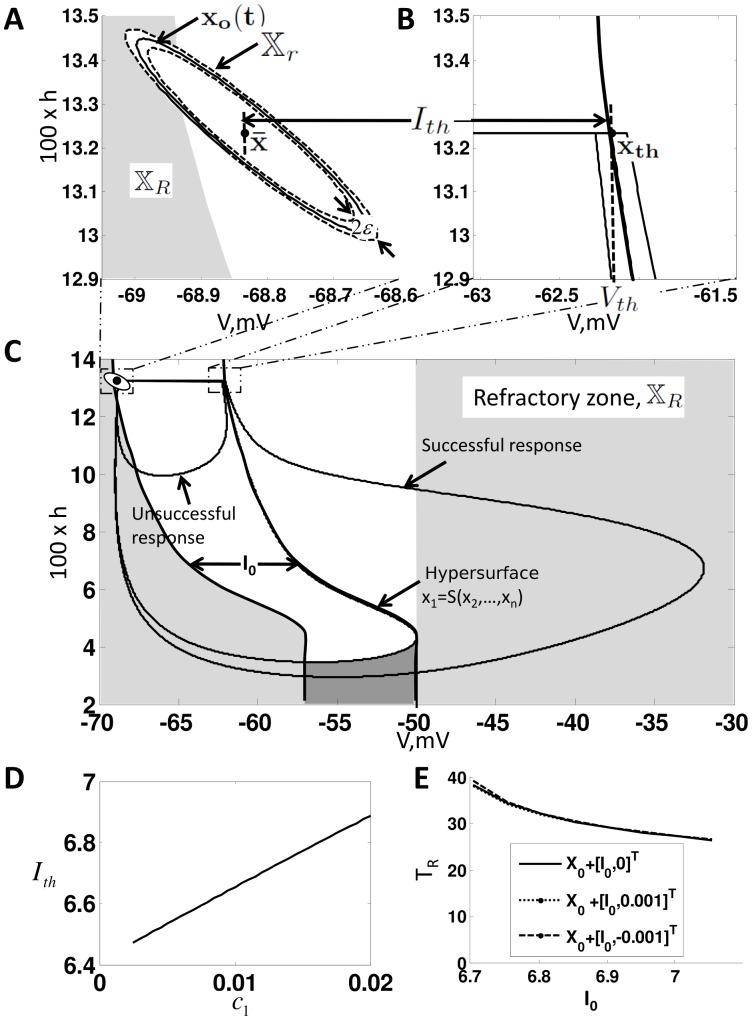
Properties properties of 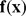 **.** (**A**) Illustrates the equilibrium point 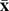, the steady state orbit 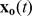 and the orbit tube, 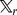, for 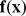 given by (3) and 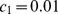. The orbit tube is shown for 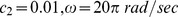. (**B**) Illustrates 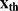, the threshold voltage 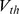 and threshold current 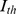. Note that these parameters are defined by the undriven system (9). (**C**) Illustrates the critical hypersurface 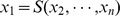, a successful response trajectory, an unsuccessful response trajectory, and the refractory zone, 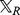 for the undriven system (9). The time it takes for the solution to leave 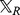 after generating a successful response is called the refractory period, 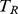. Note that refractory zone depends on 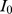 and therefore 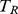 also depends on 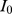. Additionally, note that the region shaded in the darker grey is also in the refractory zone, because if 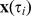 is in this region then 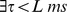 such that 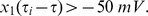 Therefore, a successful response cannot be generated if 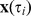 is in this region by definition. (**D**) Dependence of 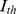 on 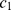. Note that 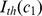 is approximately a straight line with slope 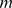, i.e 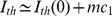. (**E**) Illustrates 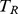 vs 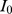 and 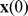.

This further implies that if a small periodic modulating input is applied to a stable neuron (4), 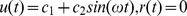, then after a sufficient amount of time the system's state vector will lie within a small neighbourhood of the equilibrium point. However, the state vector never reaches 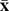 due to the time varying modulating input. The trajectory of the state in this neighbourhood can be solved using linearization methods and is periodic as we will show later. We define this periodic trajectory as **the steady state orbit** of a stable neuron, 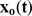. See Figure 2 A.

Next, we define 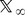 as the collection of all points in the steady state orbit. If the initial state of the system 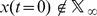 then 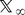 is not achievable in finite time. Therefore, we relax our definition to the collection of all points inside a tube of 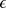 thickness around the steady state orbit, and define this tube as the set 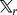, i.e.
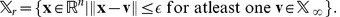
(10) An illustration of equilibrium point 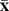, steady state orbit 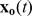 and the orbit tube, 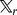 is shown in Figure 2 A.

2. **Threshold behaviour**: To define threshold behaviour of a neuron, we first define a “successful response”. A successful response at time 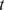 is a change in 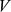 such that 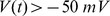 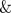 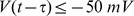 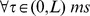. Note that both a single spike or a burst of spikes, with intra burst interval less than 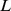 ms, are counted as a single successful response under this definition. We use this definition so that we can extend our analysis to bursty neurons characterized by higher order models.

Now, we state the following Lemma which defines the critical hypersurface.

**Figure 3:**
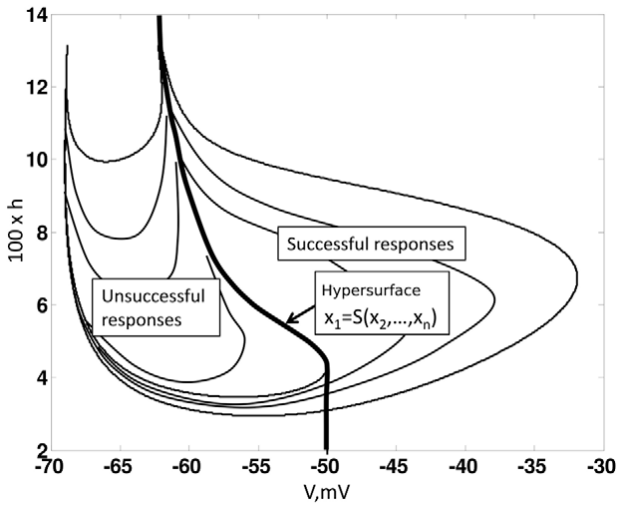
Threshold. Illustrates the critical hypersurface 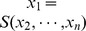, which defines the threshold for a successful response.(9) generates a successful response for any initial condition that is to the right of the hypersurface i.e. 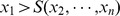. Whereas, any initial condition to the left of the hypersurface results in unsuccessful response.

**Lemma 1:** Given an 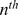 order system (9), there exists a **critical hypersurface** of the system, 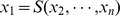 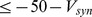, such that 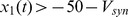 if and only if 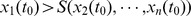 for some 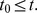 That is, the neuron only generates a successful response if the voltage crosses the critical hypersurface (see Figure 2 C and 3).

We leave a formal proof to the reader. Essentially, by definition of 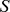, one can show that the solution to (9) always moves away from 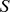, unless it is on 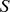 (see Figure 3). This means that at least one of the eigenvalues of 
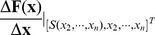
 has a positive real part. This threshold property is also used in other studies [31].

Now, we define 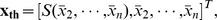 which is a point on the critical hypersurface. Note that 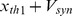, is the traditional threshold voltage 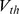 that people refer to for neurons [31]–[33]. In [31] it has been shown that spike threshold is influenced by ion channel activation/inactivation and synaptic conductance. In our case, the threshold 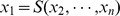 shows the same behavior as it is a function of the availability of activation/inactivation gates. The effect of time varying synaptic conductance is not captured by the hypersurface 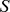. However, we used linearization methods from systems theory in section “Response in 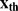 Neighbourhood Under 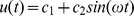
” to include this effect. This yields a time varying threshold. Although we never explicitly deal with time varying threshold, it is implicit in our analysis. Finally, we define the **threshold current,** 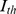, such that 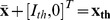. Note, by definition both 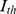 and 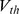 have the same units and hence can be added.

Illustrations of a successful response, unsuccessful response, the critical hypersurface 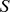, 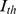, 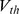, 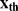 are shown in Figure 2 B, C, for a second order system. Note that, 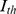 and 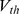 are functions of 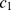, since different values of 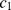 result in different 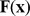 and hence different 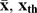. Figure 2 D, plots how 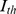 varies with 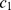 for 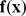 given by (3). 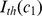 is essentially a linear function with slope 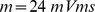, i.e 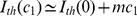.

3. **Refractory period**: Most neurons may generate a successful response when they are depolarized. However, they are unable to generate a successful response immediately after generating one. The duration for which they cannot generate a second successful response is called a refractory period [34]. This is because when a neuron returns back to its equilibrium point after generating a successful response, it becomes hyperpolarized, requiring extra depolarization to generate a new successful response. Additionally, due to inactivation of sodium and calcium ion gates, extra depolarization is required for the state to cross 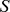 and hence generate a successful response. This extra depolarization results in an unsuccessful response soon after a successful response.

We define the **refractory zone**, 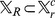 as the region such that if 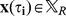, the neuron of type (4) (with 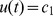) cannot generate a successful response on the arrival of a pulse in 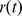 with height 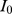 at time 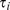. Note that 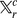 is the complement of 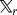.The time spent in this zone after a successful response is the **refractory period**, 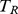. Note that, 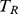 is not an absolute refractory period as a stronger depolarization event may result in a successful response even if 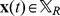.

In Figure 2 C, we illustrate 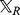 for a second order system with 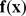 given by (3) and 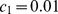. For this system, 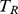 decreases with 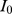, as shown in Figure 2 E. Note that 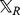 and 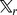 are disjoint sets by definition.

### Relay Reliability

Before we define relay reliability, we first define a *relayed pulse*. A relayed pulse is a successful response, 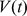, that occurs within 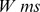 after a pulse in the driving input, 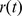. See Figure S2 (Supplementary Material). Let,
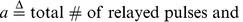
(11a)

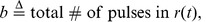
(11b) then the empirical reliability is defined as:
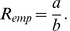
(12)


This definition of reliability is similar to the one defined in [15] and is not meaningful if 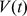 spikes without a pulse in 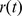. But since our neuron is a stable neuron, this will never happen. In the limit that we observe the neuron for an infinite amount of time, the empirical reliability converges to
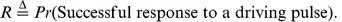
(13)


Let us define events
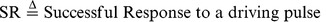
(14a)

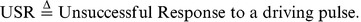
(14b)


We then see that
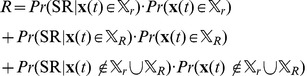
(15)


Here we have used the total probability law and the definition of conditional probability [35] to go from (13) to (15). Because we cannot generate a spike in the refractory zone, 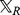, we get that
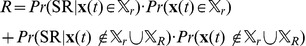
(16)


For most neurons, the dynamics of the first component of the state, 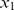, are faster than the other states in the region 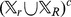, see Figure 2 C. Therefore, when 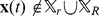, it returns to 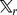 only if it is close to 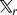, otherwise it returns to 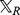. The return process to 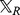 is much faster as compared to the return process to 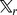, due to slower dynamics arising near 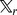. Therefore, when 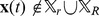, it spends most of its time close to 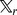, and hence we assume that the 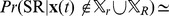 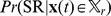. Furthermore, since the 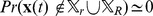, this assumption does not affect our results much. We will convince the reader that these assumptions are mild in the results section. Essentially, we will show that our reliability expressions under these assumptions match well to numerically computed curves for different relay neurons. Finally, since 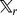 and 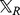 are disjoint sets, we get:
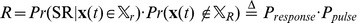
(17)


Although not explicitly in (17), relay reliability is a function of the driving input parameters, 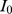 and 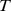, the modulating input parameters, 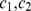 and 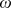 and the neuron's dynamics (i.e. model parameters) denoted by 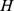. In the next sections, we compute closed-form approximations of lower and upper bounds of reliability as a function of 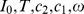 and 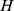, by computing 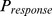 and bounds on 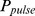.

### Calculation of 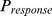


To compute 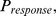 we first find a solution for the orbit tube 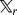 and then find a solution for the response to a driving pulse given the state starts in 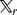. This solution shows us when the neuron generates a successful response. We later use this information to compute 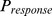.

#### The orbit tube: Response to 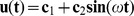 and 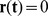


Here, we examine the state vector response to a periodic modulating input when no driving input is applied.

The solution to (4) in the orbit tube is given by its steady state solution with 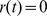. This steady state solution can be approximated using linearization (4) and linear time invariant (LTI) systems theory. Specifically, we linearize (4) about the nominal solution 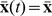 given the nominal input 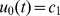. Now, if the input is perturbed such that 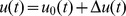 and the initial condition is perturbed such that 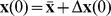, the state trajectory will also be perturbed to 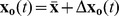. When we substitute these values and perform a first order Taylor series expansion of (4) about the nominal solution and nominal input, we get:
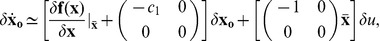
(18) which can be equivalently written as:
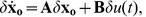
(19) where
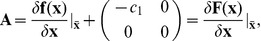
(20a)

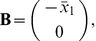
(20b)

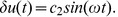
(20c)


The solution to (19) with 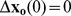, in the Laplace domain [36] is
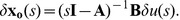
(21)


Substituting the laplace transform of 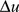 from (20) and defining
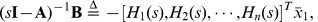
(22) we get:
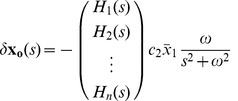
(23)


From (23), one can compute the steady state solution of (19) by taking inverse Laplace transform of (23) and taking the limit 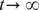. This gives:
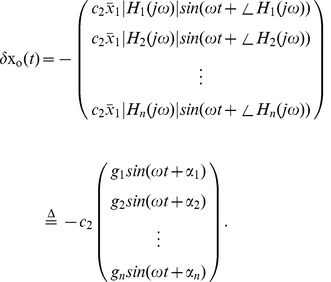
(24)


Here, 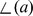 denotes the angle of complex number 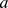. Note that 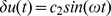 for 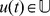. (19) approximates (4) in steady state when 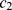 is small, which is always the case by definition of 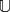. Also note that we will get the same steady state response even if 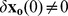. Using (24), we can write the steady state solution of (4) as:
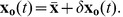
(25)


**Figure 4:**
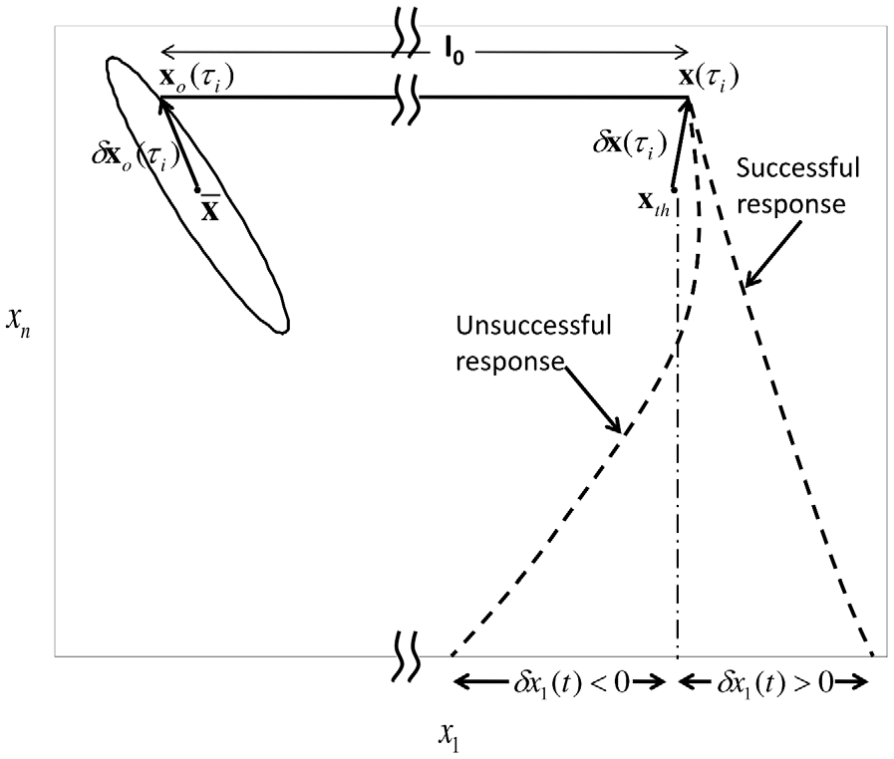
Calculation of 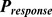 **.** Illustrates 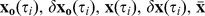 and 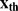. When an 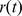 pulse arrives, the solution jumps from 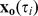 to 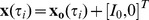. Now, whether the neuron generates a successful response or not is governed by the local dynamics. Therefore, we linearize (4) about 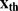 to analyze the behaviour of 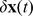 for 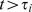. If a successful response is generated, 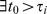 such that 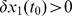 else if an unsuccessful response is generated 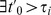 such that 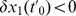.

Now we can find the orbit tube using its definition. Figure 2 A, 4, plots the steady state orbit for a second order stable neuron with 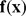 given by (3).

#### Response to 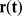 pulses and 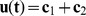 in the orbit tube

We now examine the neuron's response to a driving input pulse when the solution is in the orbit tube. It is straightforward to see how a 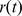 pulse affects the solution trajectory. Suppose that the state vector is at the 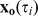 and at some time 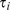, when the driving signal generates a pulse, i.e., 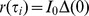. Then, the state vector “jumps” out of the orbit tube, to the point, 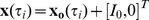 (see Figure 4). This is shown by direct integration of (4), on the time interval 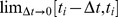. Now, three cases arise:

1. If 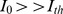, then 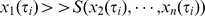 and therefore the neuron always generates a successful response.
2. If 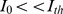, then 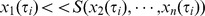 and therefore the neuron never generates a successful response.
3. If 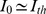, then 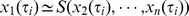 or equivalently 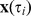 lies in the neighbourhood of 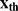. This case is biologically interesting as only for this case does the modulating input control relay reliability of the neuron. To determine whether the neuron generates a successful response or not in this case, we need to know the behaviour of the system in the neighbourhood of 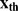.

#### Response in 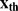 neighbourhood under 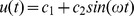


To approximate the response of the system in the neighbourhood of 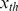, we first linearize (4) about the nominal solution 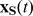 (here 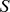 stands for the critical curve 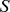) given the initial condition 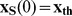 and nominal input 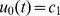. Now, if the nominal input is perturbed such that 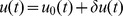 and the initial condition is perturbed such that 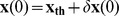, the state trajectory will also be perturbed to 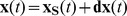. Note that in our case 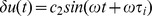 and 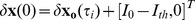. When we substitute these values and perform a first order Taylor series expansion of (4) about the nominal solution and nominal input, we get:
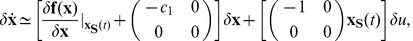
(26)


In the neighbourhood of 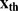 this system can be further approximated to:
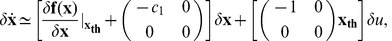
(27) which can be equivalently written as:
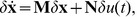
(28) where
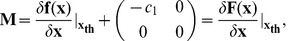
(29a)

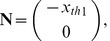
(29b)

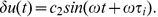
(29c)


We later show that this linear approximation does not significantly impact our expression for relay reliability of the neuron, as the numerically computed reliability fits the analytically derived curve well.

The solution to (28) is:
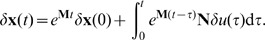
(30)


Using the eigenvalue decomposition [37] of M, such that 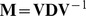, 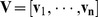, with each 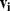 a right eigenvector, 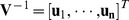 with each 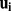 a left eigenvector and 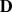 is a diagonal matrix with eigenvalues 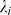 at the diagonal arranged in descending order without loss of generality, we get that
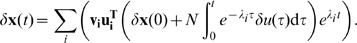
(31)


Note that for most stable neurons of interest, all the eigenvalues of matrix 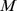 are real. Therefore, we assume real eigenvalues for an easier read (a more messy expression can also be derived for complex eigenvalues). Recall, by the properties of 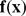, that the trajectories of (4) divert away from 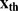, therefore 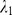 must be positive.

Now, if the neuron does not generate a successful response, 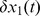 will eventually become negative. On the other hand, if it generates a successful response, then 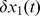 will become positive after a sufficient amount of time (see Figure 4). The direction in which 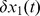 eventually moves is decided by the sign of the **first component** of the coefficient of 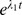. Therefore, the neuron generates a successful response if and only if
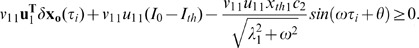
(32a)

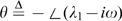
(32b)


Note that we substituted 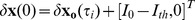 and 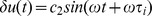 in (31) and integrated it to get (32). Now, we substitute 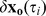 from (24) into (32) and get:
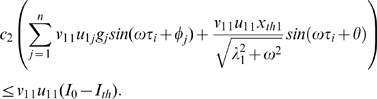
(33a)


This equation can be written as
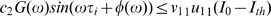
(34) where
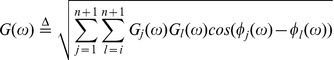
(35a)

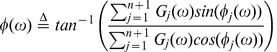
(35b)

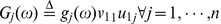
(35c)

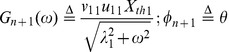
(35d)


From (34), we see that the neuron generates a successful response if and only if
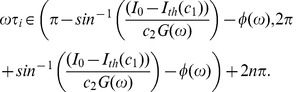
(36)


Finally, we can use (36) to calculate 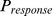, which is the fraction of the time in the orbit tube that the neuron spent in the interval in (36). This is the length of the interval divided by 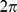. Therefore,
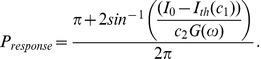
(37)


### Calculation of Bounds on 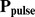


In this section, we compute 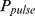 in (17) to ultimately obtain an expression for 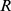. Since a driving pulse that arrives at time 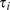 can only result in either a successful response or an unsuccessful response, we can equivalently write the definition of 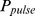 as:
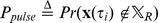
(38a)

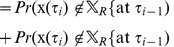
(38b)

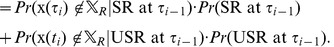
(38c)


Here, we have used the law of total probability and the definition of conditional probability [35] to arrive at (38c). We know that after a successful response at 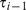, the system state 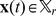, only for 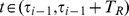. Therefore
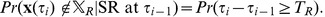
(39) Similarly, if 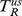 denotes time spent in refractory zone after unsuccessful response, then we get:
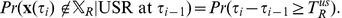
(40) Now by combining (13), (38c), (39) and (40) we get:
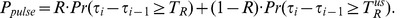
(41)


Since 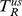 has a complicated dependence on the input and model parameters, it is difficult to calculate 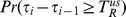. However, it is certain that 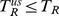. This implies that 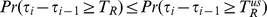, by properties of cumulative distributive functions [35]. Therefore, we get the following bounds:
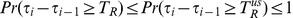
(42) Putting (41) and (42) together, we get:
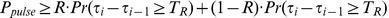
(43a)

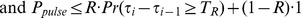
(43b)

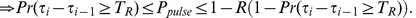
(43c) Now, we calculate 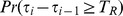. Recall that the inter pulse intervals of 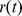, 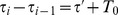, here 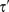 is generated from an exponential distribution and 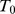 is the refractory period. Therefore:
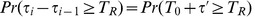
(44a)

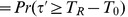
(44b)

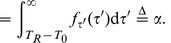
(44c) It can be easily shown that:
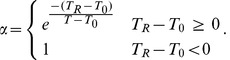
(45)



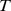
 is the average inter pulse interval, 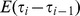. Finally, by combining (43c) and (44) we get:
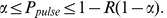
(46a)


### Calculation of Bounds on 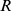


Now we compute bounds on relay reliability i.e 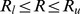. Recall that:
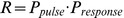
(47a)

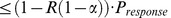
(47b)

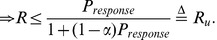
(47c)


Similarly, we can write lower bound on reliability as:
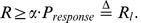
(48)


Combining (47) and (48) we get:
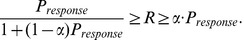
(49)


From (49) and (44), one can see that if 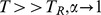, which makes 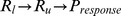. This result is intuitive because if pulses in 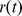 occur at a slow rate, then the solution of (4) has enough time to return to the orbit tube after each pulse. Therefore, 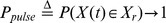 and 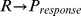.

Another interesting case emerges if 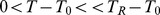. In this case 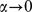 and 
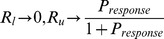
. This case has two interesting extremes: 1. 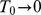, making 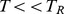, 2. 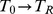, and both 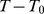 and 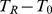 approach 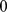. In case 1, an average a 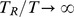 number of pulses occur in the 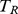 time interval after a successful response. All of these pulses generate unsuccessful responses because the system state is inside 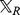 during this interval. Therefore, for each successful response, we get 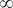 unsuccessful responses making 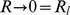. However, in the second case, exactly one pulse occurs during the 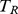 period after a successful response. Therefore, for every successful response we get at least 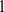 unsuccessful response. Now, if 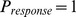, we get exactly one unsuccessful response for each successful response making 
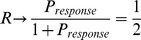
.

## Results

In this section we verify our reliability bounds by simulating a second and third order model for a thalamic relay neuron.

### 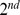 Order Model of a Thalamic Neuron

**Figure 5:**
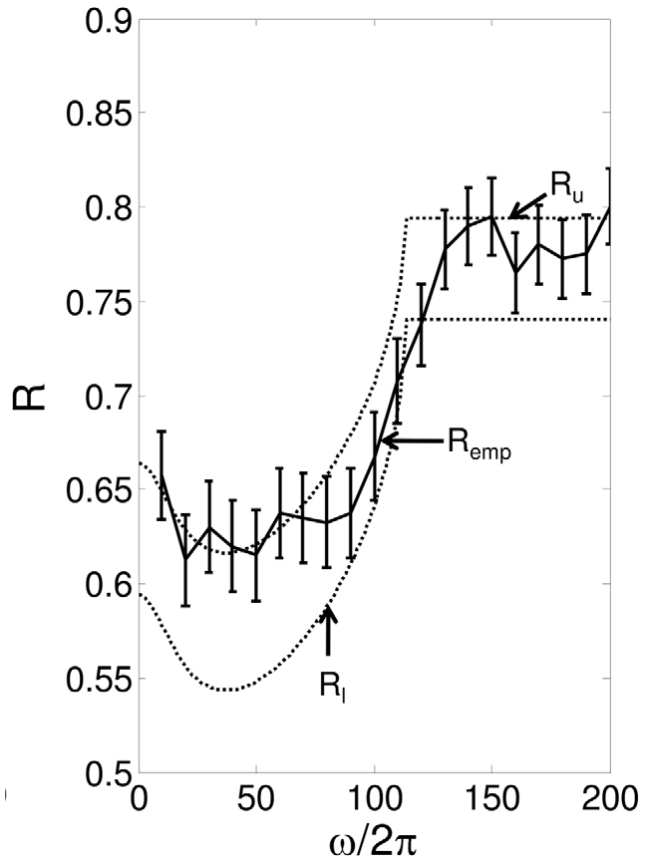
R vs 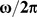 **.** Plots the theoretical and numerically computed reliability as a function of 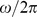, with 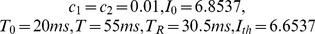. The dotted lines are the lower and upper bounds on reliability from the (48) and (47), respectively. The solid line is 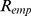 calculated by running simulations of (1), and the error bars indicate 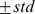.

**Figure 6:**
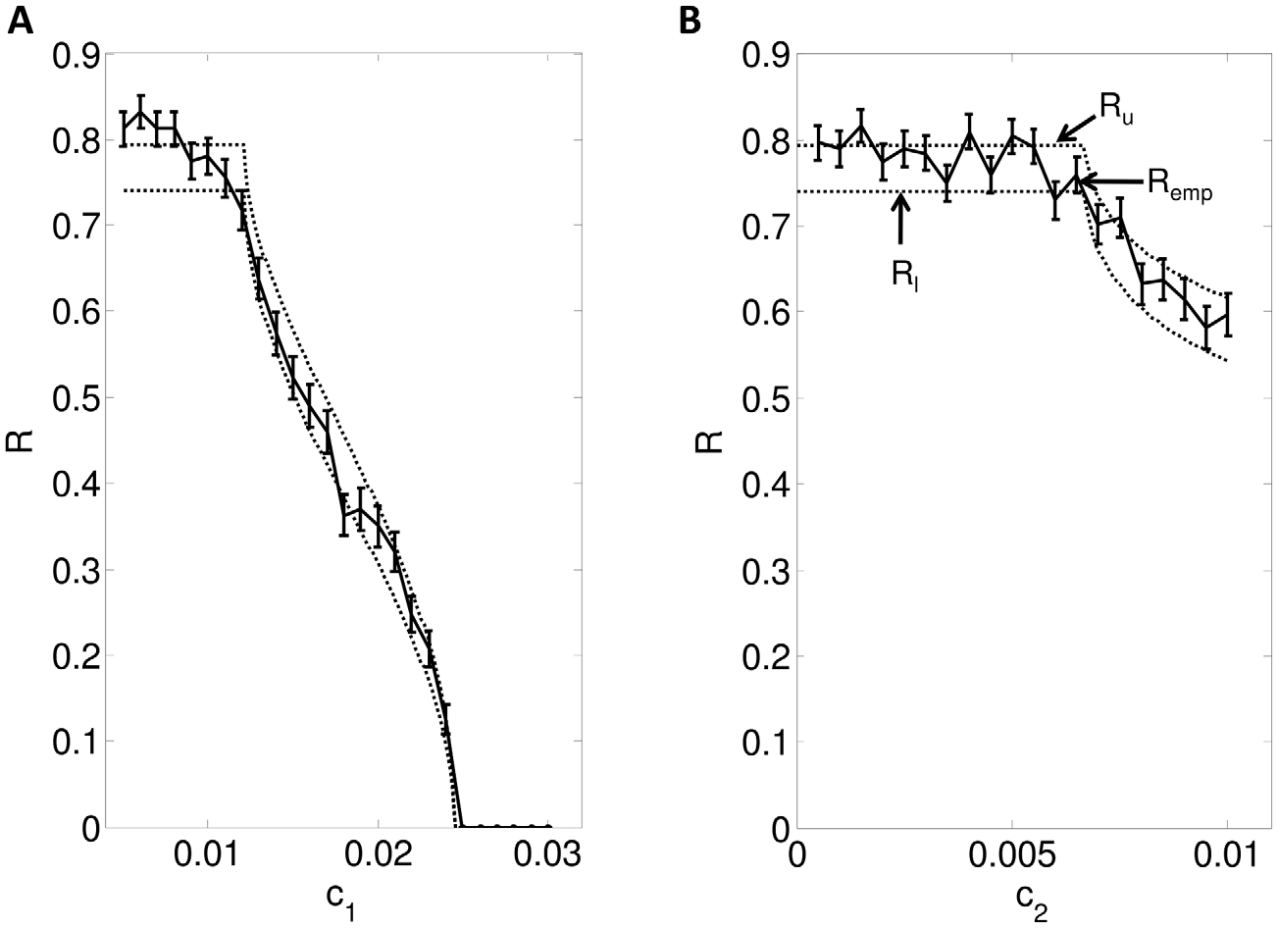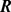 vs 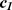 and 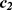 - A. Plots the theoretical and numerically computed reliability as a function of 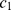, with 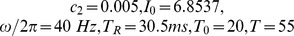. B. Plots the theoretical and numerically computed reliability as a function of 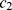 with 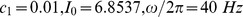, 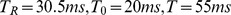. The dotted lines are the lower and upper bounds on reliability from the (48) and (47), respectively. The solid line is 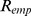 calculated by running simulations of (4), and the error bars indicate 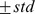.

In Figure 5, we plot 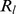 and 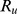 vs 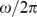 for 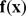 given by (3) with 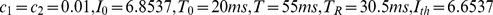, and superimpose it with a numerically obtained curve through simulation of the original model (1). 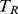 is estimated by doing repeated simulations on (4) with 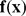 given by (3), 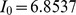 and 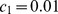. We see that empirical reliability plus and minus its standard deviation are essentially within bounds 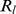 and 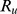. From Figure 5 B, we see that 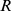 increases with the frequency of the modulating input, 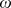. In Figure 6 A, we plot 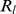 and 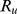 vs 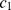 for 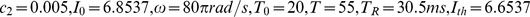, along with empirical reliability computed numerically. We see that reliability decreases as 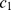 (i.e. the mean value of modulating input) increases. In Figure 6 B, we plot 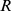 vs 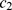 for 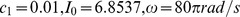, 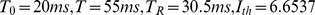. Reliability again decreases as 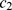 increases.

### Dependence On Model Parameters

The dependence of reliability on the cell's input parameters is explicit in our bounds. However, dependence of reliability on the model parameters is captured implicitly by the gain 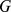, 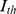 and 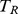. The refractory period, 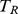, is well studied in literature and depends on inactivation gate time constants [38]. Therefore, in this section we discuss how the gain 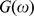 and 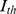 depends on the properties of a relay neuron membrane dynamics.

**Figure 7:**
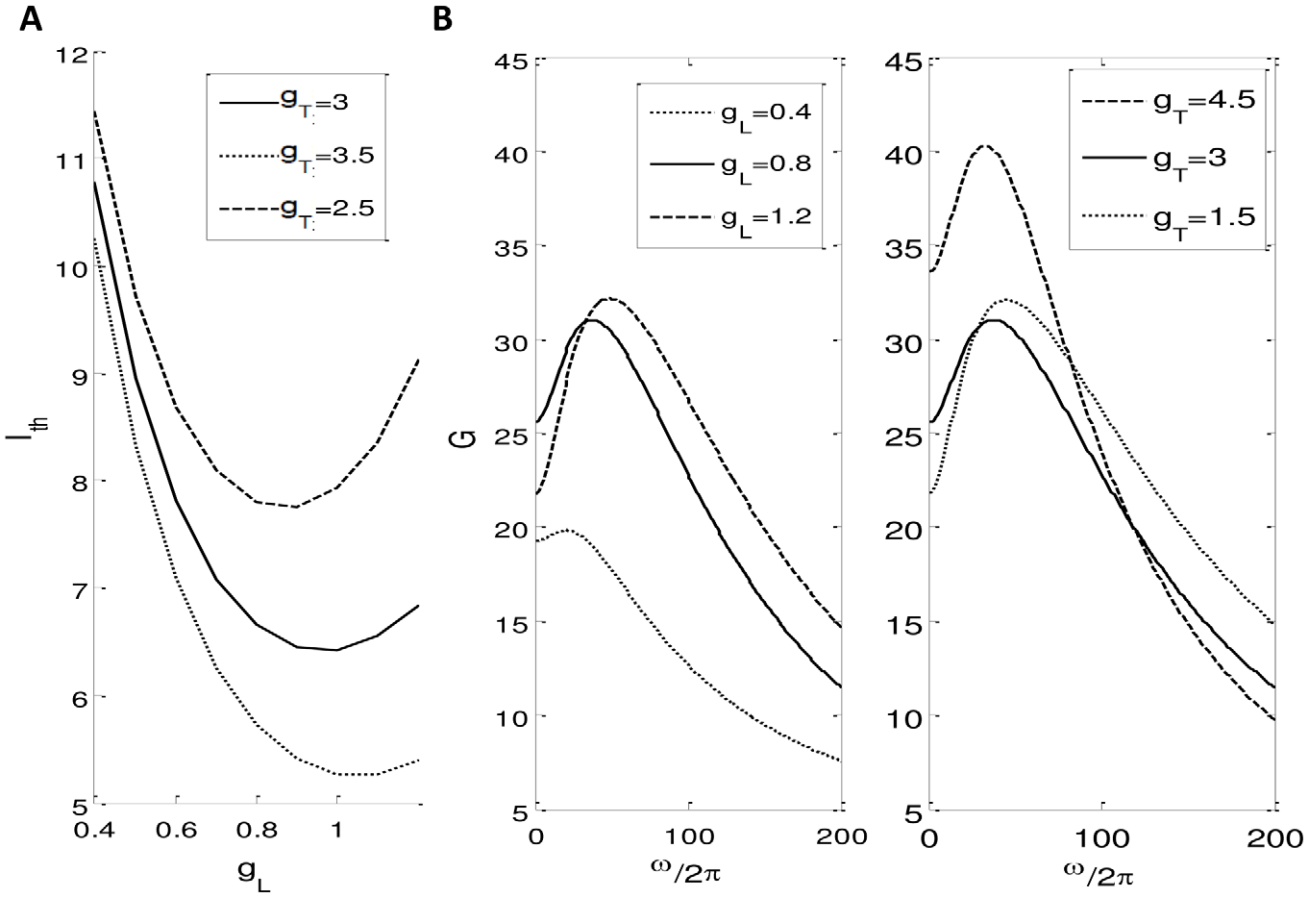
Dependence of 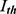 **and** 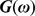 **on model parameters.** **A.** Plots 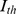 as a function of 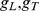 **B.** 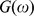 (see (35) versus 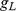 and 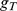. Note that 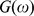 depends largely upon 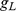, whereas its dependence upon 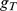 is minimal. 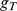 changes the maximum value of 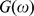 but does not effect it much in the high frequency range.

In Figure 7 A, we plot 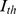 vs conductances 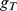 and 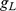. We see that 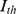 first decreases with increasing 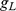 and then increases forming a parabola. Furthermore, with increasing 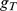, 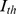 decreases. In Figure 7 B, we plot the dependence of the gain 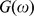 on 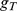 and 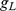. 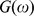 is essentially a low pass filter whose amplitude decreases as frequency increases. Consequently, reliability increases with frequency (see (49)). From the Figure, we can see that the gain, 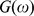, in the high frequency range (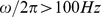) increases with 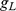 and decreases with 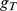. For lower frequencies, 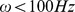, 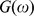 has a complex dependence on 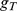 & 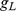. This is an important result as we can increase/decrease reliability of the relay neurons by increasing/decreasing T-type 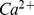 or leak channel conductances which can be further used to treat diseases such as Parkinson's disease (see discussion).

### A 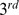 Order Model of a Thalamic Neuron

In this section, we will apply (49) to a third order model of a thalamic relay neuron. In this case, the parametrs 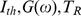 in the equation are computed from the third order model.

**Figure 8:**
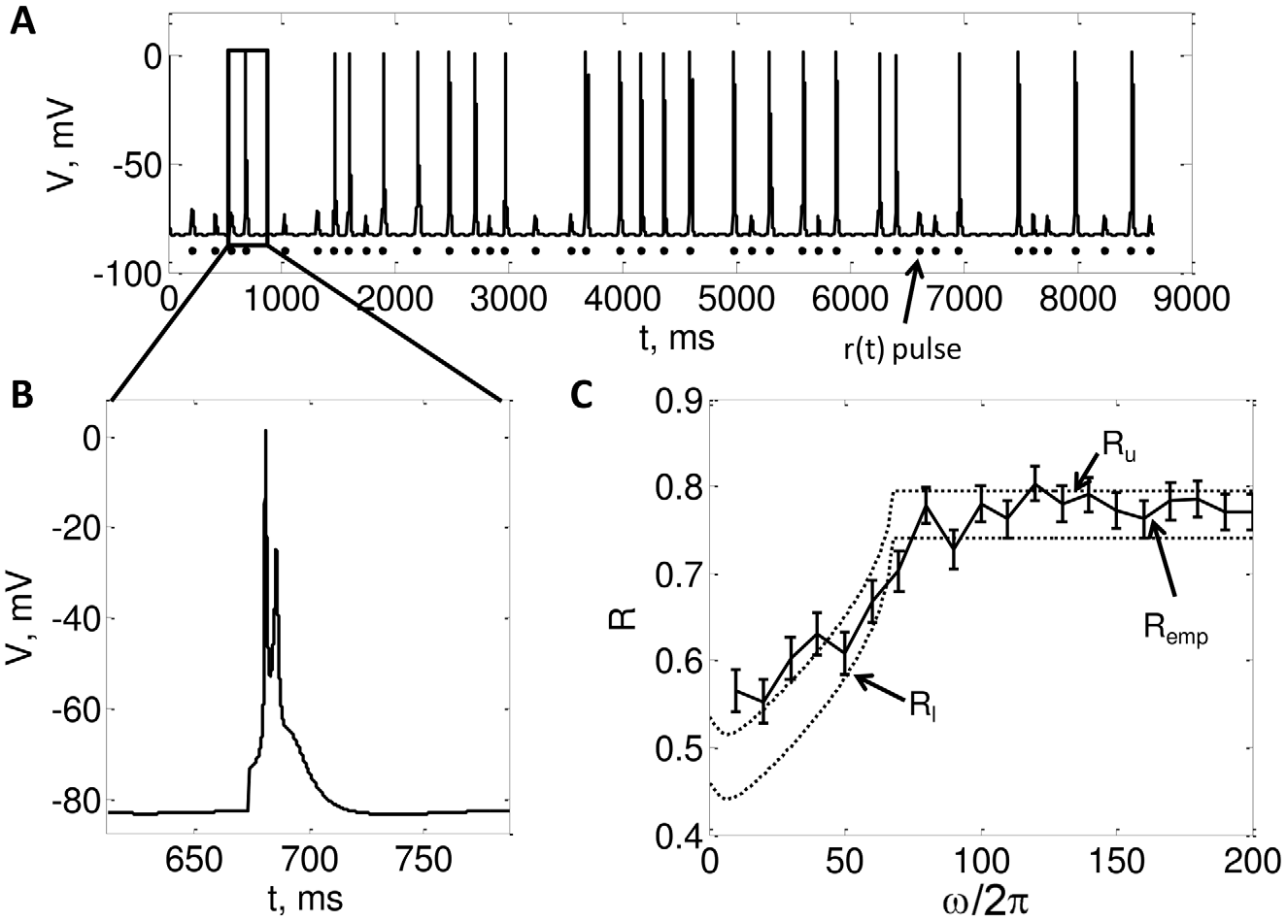
A. Voltage profile of the 3rd order model in the bursting mode (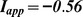 **) B. zoomed in view of a burst C.** 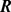 **vs** 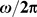 **for the** 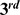 **order model.** In this Figure, we illustrate the results from a 3rd order model of a thalamic neuron. **A.** Plots the voltage profile obtained from the model in response to pulses in 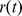. Note that each pulse in 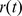 either generates a burst of spikes or does not spike at all. **B.** Zoomed in view of a burst. **C.** Plots the theoretical and numerically computed reliability as a function of 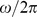, with 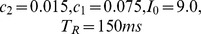,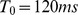,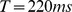. The dotted lines are the lower and upper bounds on reliability from the (48) and (47), respectively. The solid line is plots 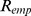 calculated by running simulations of (4), and the error bars indicate 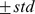. We estimated 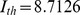 as the minimum height of a 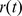 pulse that makes the neuron generate a successful response.

**Figure 9:**
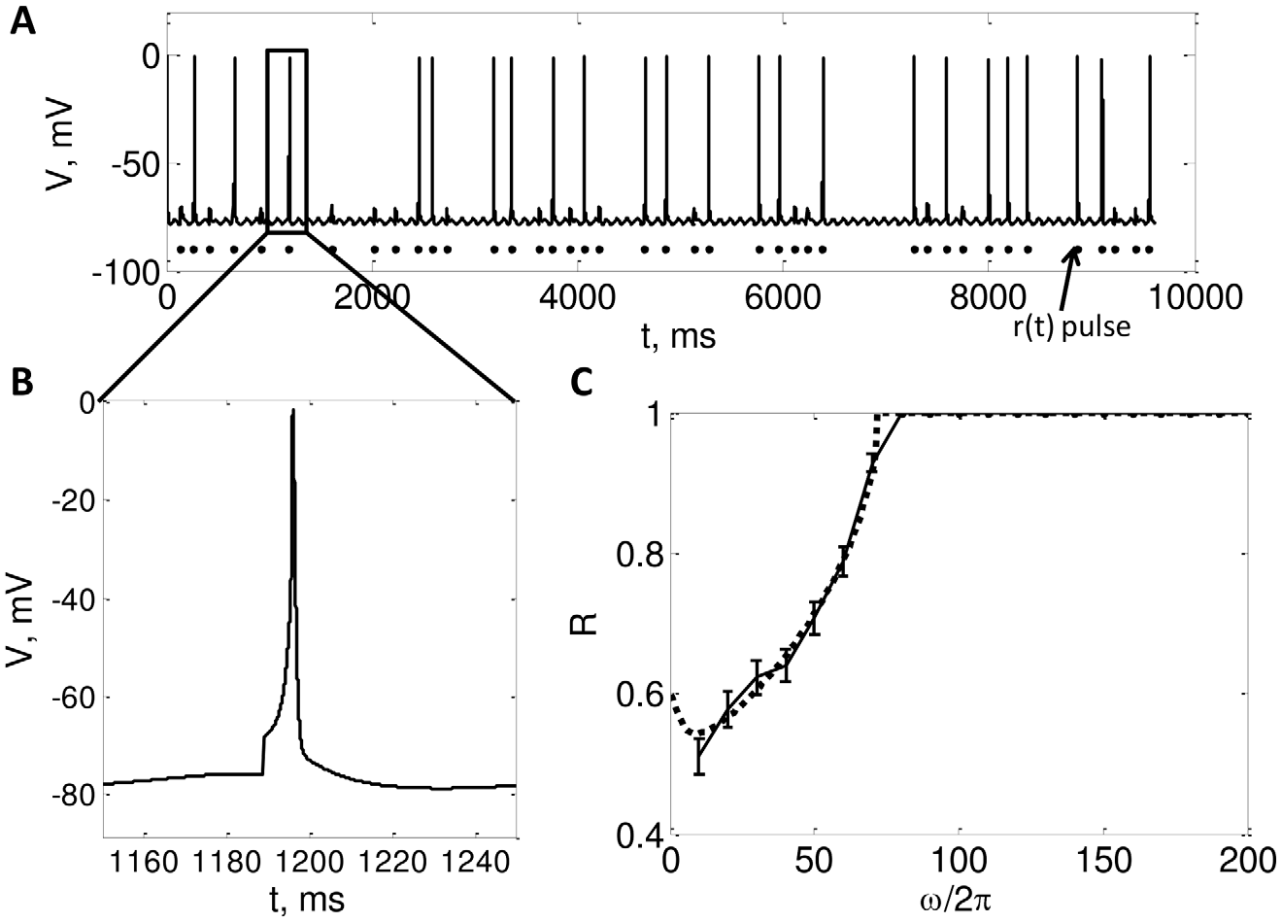
A. Voltage profile of the 3rd order model in the tonic mode (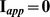 **) B. zoomed in view of a spike C.** 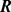 **vs** 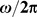 **for the** 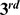 **order model.** In this Figure we illustrate the results from a 3rd order model of a thalamic neuron. **A.** Plots the voltage profile obtained from the model in response to pulses in 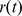. Note that each pulse in 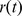 either generates a successful spike or generates unsuccessful spike. **B.** Zoomed in view of a successful spike. **C.** Plots theoretical and numerically computed reliability versus 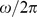, with 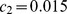, 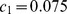, 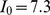, 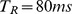, 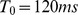, 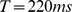. The dotted line is plotting the lower and upper bounds on reliability from the (48) and (47), respectively. Note that here 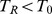, therefore 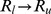. The solid line plots 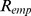 calculated by running simulations of (4), and the error bars indicate 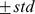. We estimated 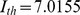 as the minimum height of a 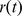 pulse that makes the neuron spike.

We chose the 3rd order thalamic model used in [15], [16], [22], which is a simplification of model used in [39], [40]. This model exhibits bursting activity in the hyperpolarized state and non bursty firing in the depolarized state. The two responses of the model for an oscillating modulating input and a Poisson driving input (inter-pulse interval is given by (7)) are shown in Figure 8 A and 9 A. The equations and parameters of the model are the same as those used in [15], [22]:
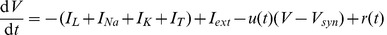
(50a)

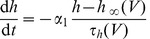
(50b)

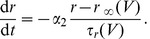
(50c)


In the (50), 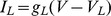, 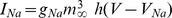, 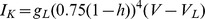 are the leak current, sodium and potassium current, respectively. 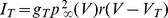 and 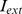 are the low threshold potassium current and external current respectively. 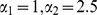 are the temperature correction factors. All the parameters used are given in Table 3.

**Table 3:** Parameters and functions for (50).

Function	Value
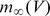	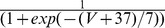
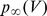	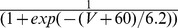
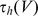	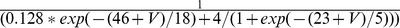
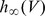	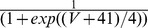
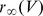	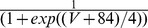
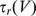	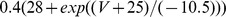
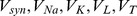	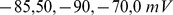
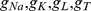	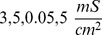

A thalamic neuron generates a single spike when depolarized in the relay mode [15], [41]. However, it generates a burst of spikes when it receives a depolarizing input when it is in a hyperpolarized state [42]. We used 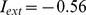, to model the hyperpolarized or bursty state. Whereas, 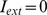 models a single spike state of thalamic neuron.

We can rewrite the (50) in the form of (4) by defining the state vector 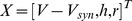 with:
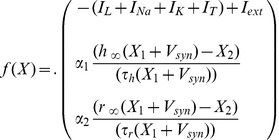
(51)


In Figure 8 A, we plot the time profile of the voltage for a bursty neuron along with a zoomed in view of the burst in Figure 8 B. Figure 8 C plots our reliability bounds (49) along with empirical reliability computed numerically through simulation of the 3rd order model. We see that our bounds predict reliability well even for a bursty neuron. Note that we consider a burst response to a pulse as a successful response.

In Figure 9 A, we plot the time profile of voltage for a non bursty neuron along with a zoomed in view of a successful spike in Figure 9 B. Figure 9 C plots our reliability bounds (49) along with empirical reliability computed numerically through simulation of the 3rd order model. Note that here 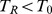 therefore 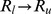. We see that our bounds predict reliability well in this case also.

In general, our analytical bounds are applicable as long as the model 1. does not generate a spike if there is no pulse in 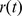, and 2. has a threshold behaviour as defined in Materials and Methods section, and 3. shows a refractory period. The second condition is true for most neurons that satisfy the first condition. Our analysis may also be extended to include neurons that spike without any driving input (see Discussion), but in this manuscript we neglect such dynamics.

## Discussion

In this manuscript, we studied the reliability of a relay neuron. A relay neuron receives two inputs: a driving input, 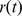, and a modulating input, 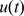. The neuron generates one output, 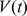, which relays 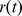 conditioned on 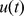. Our goal was to precisely determine how the modulating input impacts relay reliability. To calculate relay reliability, we used systems theoretic tools to derive the analytical bounds (49) on relay reliability as a function of different input and model parameters. Specifically, (49) implies that if the modulating input is of the form 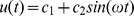, then increasing 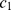 or 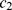 decreases reliability. However, increasing 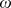 increases reliability. In addition, our reliability curve (see Figure 5) suggests that reliability first increases slowly with 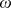 and then increases rapidly and plateaus. (49) is powerful as it characterizes the multiple dependencies of reliability on 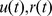 and relay neuron model parameters. Furthermore, analytic bounds from (49) contain results obtained through simulation of the 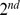 and 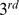 order models of a relay neuron. Our bounds captured reliability under both the depolarized and hyperpolarized states of the 3rd order neuron and shows the generality of our analysis.

### Comment on Spontaneous Firing in Relay Neurons

Our reliability bounds were calculated assuming that the relay neuron does not fire spontaneously. However, many relay neurons show spontaneous firing in the absence of any input. This spontaneous firing is usually periodic (period 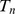) because it arises from the emergence of a limit cycle [43] and can be thought of as responses to background noise. Our analysis can therefore be extended to capture this by adding a periodic noise pulse train 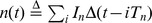 in the reference input 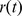, therefore the new reference input becomes:
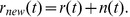
(52)


Since a successful response to a pulse in 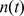 is undesirable, we must modify our definition of reliability. To do this, we assume that the arrival of a pulse in 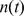 cannot coincide with an arrival of a pulse in 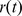 and thus successful responses to pulses in each signal are disjoint events. This leads us to define reliability as
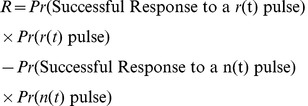
(53a)


With this approach, our analysis can be extended to spontaneously firing neurons. We believe that the reliability will approximately be bounded as:
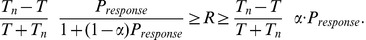
(54)


The above expression is reduced to (49) in the case 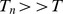 i.e the noise period is much larger than the period of the driving input. In the case when 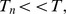 the reliability becomes negative because noise pulses occur very frequently as compared to desirable driving input pulses. This generates undesirable successful responses making reliability negative. Note that (54) is only an approximate solution for the reliability of spontaneously firing relay neurons and we leave the exact solution to this problem for the future work.

### Motor Signal Processing

**Figure 10:**
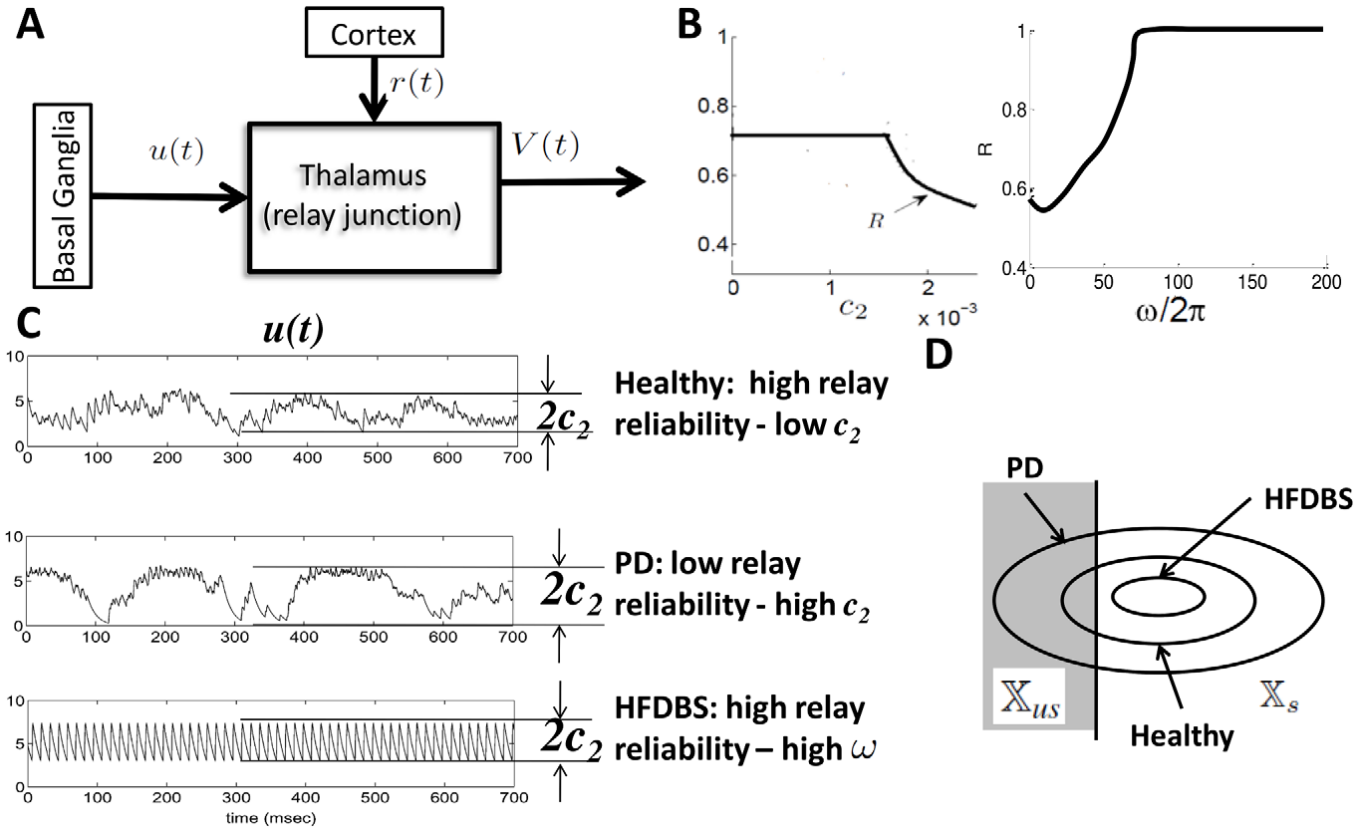
Thalamocortical loop in motor signal processing. (**A**) Simplified view of basal ganglia thalamo-cortical motor signal processing. Sensorimotor cortex generates the driving input and projects to the motor thalamus. The thalamus relay of cortical input is modulated by the basal ganglia (BG). (**B**) Relay reliability curves computed from our analysis as a function of 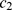 and 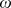 from (49). (**C**) Simulations of 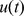 (basal ganglia output) from the computational study [15] for the Healthy, PD and PD with high frequency deep brain stimulation (HFDBS) cases. As we can see in the healthy case, the amplitude of the BG output, 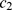, is smaller compared to the PD BG output, resulting in a higher relay reliability. HFDBS increases the frequency, 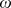, of the BG output, resulting in a higher relay reliability. (**D**) Intuition of how reliability changes in the three cases. In PD, 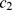 is larger, therefore, the diameter of the orbit tube is larger compared to the orbit tube for healthy. This results in more time spent in the unsuccessful response region 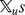, which leads to poor reliability. In contrast, in PD case with HFDBS applied, 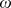 is larger and the gains 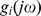 decrease, which generates a smaller orbit tube. In this case, the state spends more time in the successful response region 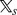 of the orbit tube, resulting in high reliability.

In the motor circuit, thalamocortical neurons receive a driving input from the motor cortex and a modulating input from the GPi segment in the basal ganglia (BG). See Figure 10 A. The function of the GPi input is hypothesized to enable/disable thalamic cells to relay cortical stimuli related to movement when movement is intended/not intended [14]. This is consistent with evidence that the BG both inhibits unwanted movements and enables intended movements in a timely manner [12], [13]. This GPi modulated thalamic relay ultimately enables reliable transfer of information from higher cortical layers to lower layers which then command the musculoskeletal system to generate planned movements [44]. The thalamic relay hypothesis is supported by previous studies [4], [16], [22]. In [16], [22], it is shown that relay reliability computed from a data-driven computational model of a thalamic neuron is low in Parkinson's disease (PD), and high in both healthy and when therapeutic DBS is applied to the BG in PD.

Previous works emphasize the inhibitory projections from GPi to motor thalamus [45]–[48]. They argue that when movements are intended/not intended, appropriate task-related GPi neurons decrease/increase their firing rates. This in turn disinhibits/inhibits thalamus and consequently enables/disables thalamic relay, respectively. Our analysis as well as recent experimental observations show that the story is a bit more complicated. GPi firing rates alone may not be the mechanism for thalamic relay, rather, the dynamics of the GPi activity control thalamic relay. In particular, it appears that the *oscillatory dynamics* of GPi activity control relay. Our relay bounds predict that if one intends to move, then the GPi neurons that project to motor thalamus should initially generate LFP activity that has prominent low frequency oscillations which allows the subject to remain idle, and then generate activity that has prominent high frequency oscillations which allows the subject to plan an intended movement and then move.

We first discuss how our analysis concurs with observations obtained from a computational model of the motor circuit that characterizes neural activity dynamics in the BG and motor thalamus in health and in PD with and without therapeutic DBS. The computational model simulates neural activity when movements are planned and hence when motor thalamus should relay information from the cortex at all simulated times. We then discuss how our relay bounds accurately predict how GPi activity recorded from two healthy primates modulates during a structured behavioral task that forces an idle phase, and a planning phase during each task trial.

#### Predicting data from a computational model

In PD, the GPi input to thalamus becomes pathological and prevents the thalamus from properly relaying information back to the cortex. In particular, people have observed pathological 10–30 Hz beta rhythms and synchronization emerging throughout the BG in PD [49]–[52]. High frequency DBS (HFDBS) modulates activity in the BG structures, including GPi, and may restore thalamic reliability leading to clinically observed reversal of symptoms in PD [51], [53].

To better understand how HFDBS may restore relay reliability, we first consider a computational study [15] of basal-ganglia-thalamic neural signal processing. In [15], a biophysical-based model of multiple BG structures and motor thalamus is constructed and parameters are tuned to generate 3 states: healthy, PD and PD with HFDBS applied to the subthalamic nucleus (STN) in the BG. In Figure 10 C, we reproduce plots from this study that illustrate the simulated GPi modulating input to thalamus in the 3 states. We then discuss how our reliability bounds predict what is observed in these simulations.

- According to the computational model in [15], in the healthy case, relay reliability is high. When we look at the simulated Gpi activity, the amplitude of the GPi modulating input, 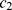, is small enough to generate reliable thalamic relay of cortical inputs in accordance to our bounds (49). As Figure 10 D shows, the orbit tube is small for such a 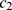, which results in less time spent in the unsuccessful response region, 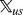. Physiologically, a small 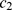 may be due GPi neurons being uncorrelated so that when they add they do not produce large LFP amplitudes. Gpi neurons have been observed to be uncorrelated in healthy primates [54], [55].
- According to the computational model in [15], in PD, reliability is low. When we look at the simulated GPi activity, the amplitude of the GPi modulating input is larger than in the healthy case, which leads to a lower relay reliability at the thalamus according to our bounds (49). As Figure 10 D shows, the orbit tube is large for a larger 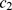 and results in more time spent in the unsuccessful response region, 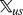. In Figure S3 (Supplementary Material), we have plotted R versus 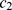 and 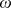. From the Figure S3, it is clear that when 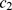 increases, reliability decreases, whereas when 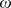 increases reliability increases. Physiologically, a large 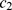 may be due to GPi neurons being synchronized so that when they add their peaks sum producing large LFP amplitudes. Synchronization of neurons in the BG has been observed in PD patients [56] and parkinsonian primates [49], [55].
- According to the computational model in [15], HFDBS applied to the PD model restores thalamic relay. When we look at the simulated GPi activity under HFDBS applied to the STN, the frequency of the GPi modulating input increases and the amplitude, 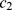 decreases and is more comparable to that in the healthy state. This combination (increased 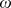 and decreased 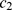) restores relay reliability according to our bounds (49). As Figure 10 D shows, the orbit tube is small for a larger 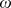 because a larger 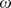 results in a smaller 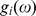
s and hence a small 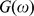. Recall that 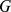 is proportional to the diameter of the orbit tube (see Figure 10 D).This results in less time spent in the unsuccessful response region, 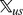. Note that the frequency of DBS is not directly related to frequency of modulating input. One can see from Figure 10 C, that modulating input frequency is only 80 Hz while HFDBS frequency is 140 Hz.

The working mechanisms of HFDBS in PD are thought to be (i) suppression of pathological beta oscillations in the BG [49], [51], and (ii) desynchronization of BG neurons [22], [57]. Our analysis accurately predicts this, but also highlights new possible therapies. For example, as discussed in section Dependence On Model Parameters, the conductance of leak channels is critical for a relay neuron because it decides the size of the orbit tube for a given 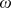. In particular, for smaller 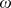, the gain 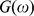 decreases as we decrease 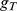, which results in increased reliability. This suggests that if we could pharmacologically decrease 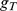, a lower frequency (hence lower power) DBS signal may be therapeutic. A low power DBS option would save battery power as well as minimize side effects associated with high power stimulation [58]–[61]. There are many ways to regulate the conductance of T-type calcium channels, reviewed in [62]. These methods include (1) hormonal regulation by dopamine, serotonin, somatostatin, opioids, ANP, and ANG II. (2) Guanine nucleotides (3) Protein kinases (4) voltage. To be target specific, these methods may require injecting the chemical directly into the thalamus.

#### Predicting data from experiments

The computational model in [15] does not capture the subject's intent. It is assumed in [15] that the subject is moving and that the sensorimotor cortex sends a driving input to a thalamic cell accordingly. In reality, a subject's motor program is coordinated in time. When a subject is idle, then the activity of GPi neurons (modulating input) should have slower oscillatory patterns according to our analysis so that the thalamus does not relay information. Furthermore, when the subject plans to move, the task-related GPi neurons should then generate more high frequency oscillations to enable relay of this movement via the thalamus. This can be understood more clearly by looking at the Figure 10 A. When the subject is idle, 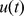 should be a low frequency signal and when the subject plans to move, 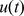 should change to a high frequency signal.

This has been observed recently, when we showed that task-related GPi neurons indeed exhibit a “crossover effect” during movement planning in two healthy macaque monkeys executing a directed hand movement task [63] (see Figure S4, Supplementary Material). Initially, when the monkey is idle, there are prominent 10–30 Hz beta oscillations in the neuronal spiking activity. Then, when a final cue is given to indicate what movement should be executed, gamma band oscillations (30–70 Hz) emerge in the spike trains of GPi, displaying the “crossover” (beta gets suppressed while gamma emerges) [63].

Futhermore, if GPi's mechanism in motor control is to modulate its oscillatory rhythms in a timely fashion as our relay analysis predicts, then the prominent beta oscillations observed in PD [49], may partially block this mechanism. That is, in PD it may be more difficult to suppress beta during movement planning as it is so prominent, leading to poor thalamic relay and poorly generated movements.

Finally, we highlight another recent study of ours that showed that when therapeutic DBS (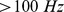) is applied to the STN of a parkinsonian and healthy primate, then the propensity of GPi neurons to spike in the gamma band increases [17]. This finding, along with the above observations, indicate that perhaps the mechanism of HFDBS is to re-enable the crossover effect in GPi (i.e. increase gamma oscillations to overcome the prominent beta oscillations) that controls thalamic relay and movements in PD.

### Visual Signal Processing

As mentioned in the Introduction, neurons in the LGN receive driving input synapses from the retina and modulating input synapses from layer 6 of the visual cortex and the brain stem. The LGN then relays the driving input to visual cortex for perception. The LGN functions as a “gatekeeper” and allows only the relevant information to go through depending on attentional demands [7], [64]. In the LGN, the spatial map of the visual field is conserved [64], [65].

Here, we hypothesize that the LGN functions as a filter of the spatial map which shows a high relay reliability in spatial areas requiring high attention and lower reliability otherwise. Our analysis suggests then that LGN neurons relaying attended areas of the visual field receive higher frequency modulating inputs as compared to LGN neurons relaying areas which are ignored. Note that the modulating input represents the synaptic background activity, which is a major contributor to LFPs and EEG recordings [10]. Therefore, the frequency content of LFPs and EEG reflect the frequency of the modulating input.

This hypothesis is supported by [8], where it was shown that the frequency of the LFPs in LGN depends on the arousal state of the cat. Specifically, they showed a prominent 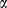 rhythm (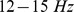) in awake and naturally behaving cats, a 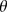 rhythm (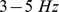) in drowsy cats and a slow rhythm (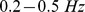) during sleep. Additionally [21], showed that, in wakeful naturally behaving cats, the spiking activity of relay-mode (non-bursty) neurons in the LGN is correlated with the phase of the alpha rhythm of the LFPs. Specifically, some neurons spike more at the peaks of the alpha wave while other neurons spike more at the valleys of the alpha rhythm. Using (36), we may be able explain why such phase locking occurs. In words, this equation says that relay neuron reliably relays the driving input only during a fixed phase interval of modulating input, and this phase interval depends on neuron membrane properties [21].

Finally, during deep sleep slow delta rhythms are observed in the EEG which are believed to be of thalamic origin [66]. This may cause even lower reliability in LGN and filter out all the visual information, resulting in deep sleep.

On the other hand, high frequency 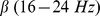 & 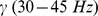 rhythms are observed during visual attentional tasks in the LFPs of cat LGN [9]. Our analysis shows that reliability increases with modulating input frequency, therefore we propose that the reliability during these tasks is greater than during natural wakeful behaviour for most LGN neurons. This results in larger relay of information which increases general productivity.

In addition to the observed relationship between the LGN LFP oscillations and attention, it has been observed that during sleep, LGN neurons become hyperpolarized [42], [67]. In our model, this means that the DC offset of the modulating input, 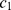, is large which decreases reliability according to our analysis. The LGN neurons relay poorly and also exhibit a bursty behaviour (see Figure 6 A and 8). The lower reliability may result in less information relay from the LGN to the visual cortex, inducing sleep whereas the bursty behaviour may only be a by-product of hyperpolarization and may have nothing to do with information suppression. This agrees with [68] where in it is shown experimentally that although all bursts combine carry lesser information than all single spikes, individual burst is more informative than a single spike in the LGN output. The information carried in the bursty mode may be critical for waking up [42].

## Supporting Information

Figure S1
**Driving and modulating inputs.** (**A**) Driving Input. Each arrow denotes a delta pulse with height 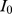. The inter-pulse interval is 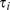, which is an exponential random variable. **B** Modulating Input. A sinusoidal wave with DC value 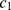, amplitude 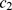 and frequency 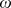.(TIFF)

Figure S2
**The relayed pulse and the non-relayed pulse.** A pulse is called a relayed pulse if it generates a successful response in 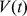 within a 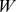 ms window, otherwise it is called an ineffective pulse.(TIFF)

Figure S3
**Reliability vs** 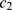 **and** 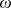 **.** Note that increasing 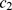 decreases reliability whereas increasing 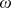 increases reliability.(TIFF)

Figure S4
**Neurons in GPi show a crossover effect during the planning phase.** In this experiment, two primates executed a directed hand-movement task. From the figure, we can see that the percentage of neurons displaying more power in gamma band compared to beta band increases just after the final command is given. This may be because the GPi output is the modulating input to the relay neurons in motor thalamus, and a increase in the frequency of the modulating input may allow a certain motor plan to be relayed back to cortex and downstream to brain stem to ultimately get executed. This figure has been taken from [63]. The thin solid and dotted lines are the 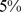 and 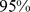 confidence bounds obtained by randomization of the spike trains.(TIFF)
