# Supplementary figures and images for: Performance Limitations of Relay Neurons
Source: PLoS Comput Biol. 2012 Aug 9;8(8):e1002626. doi: 10.1371/journal.pcbi.1002626 (PMC3415468; doi:10.1371/journal.pcbi.1002626)

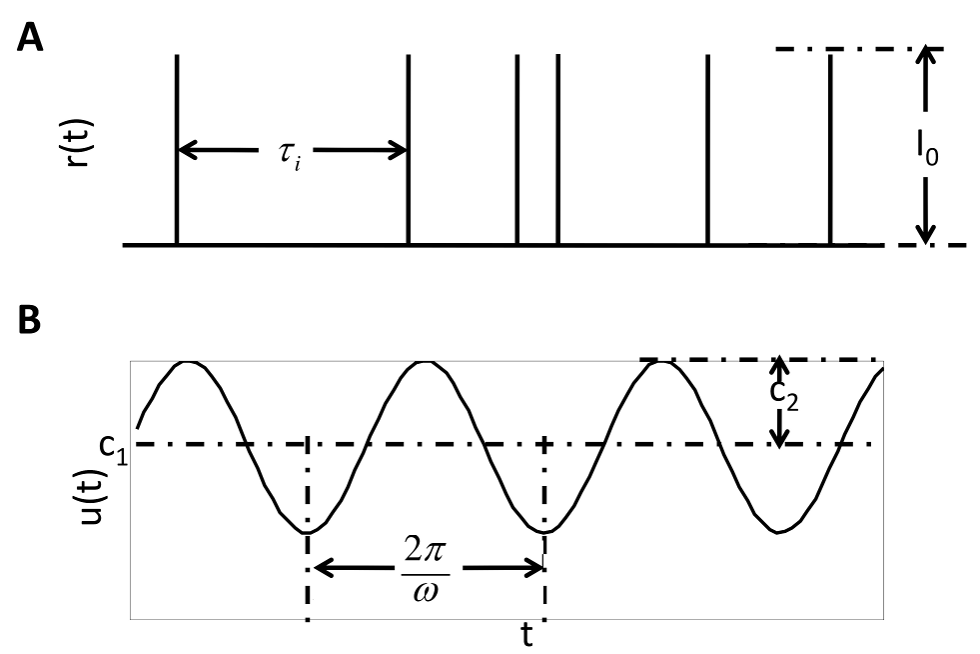

Supplement: Figure S1 — Driving and modulating inputs. (A) Driving Input. Each arrow denotes a delta pulse with height . The inter-pulse interval is , which is an exponential random variable. B Modulating Input. A sinusoidal wave with DC value , amplitude and frequency . (TIFF) [file pcbi.1002626.s001.tiff]

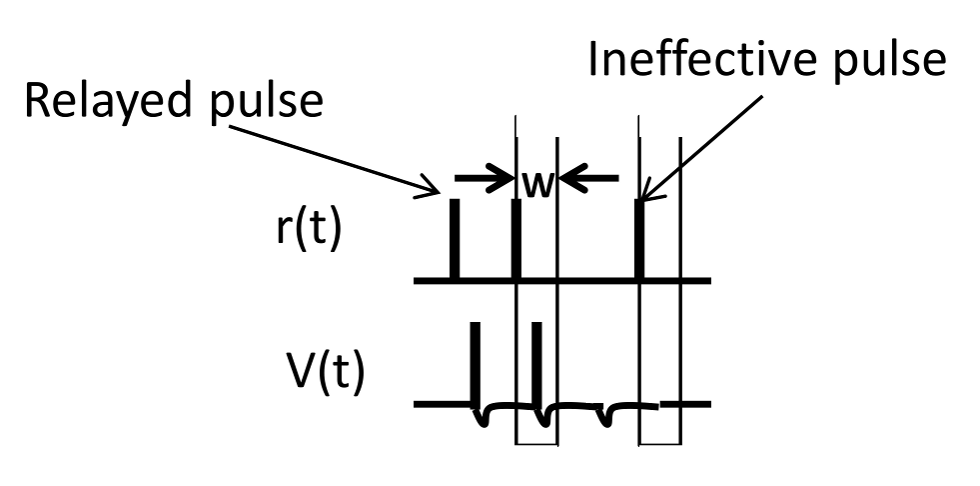

Supplement: Figure S2 — The relayed pulse and the non-relayed pulse. A pulse is called a relayed pulse if it generates a successful response in within a ms window, otherwise it is called an ineffective pulse. (TIFF) [file pcbi.1002626.s002.tiff]

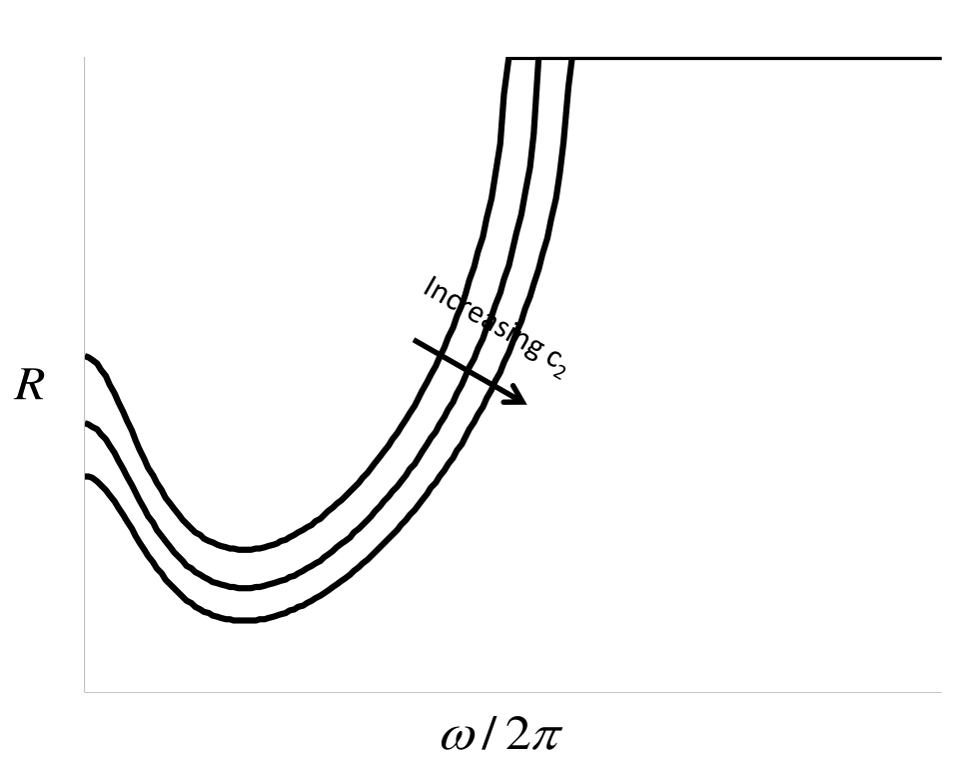

Supplement: Figure S3 — Reliability vs and . Note that increasing decreases reliability whereas increasing increases reliability. (TIFF) [file pcbi.1002626.s003.tiff]

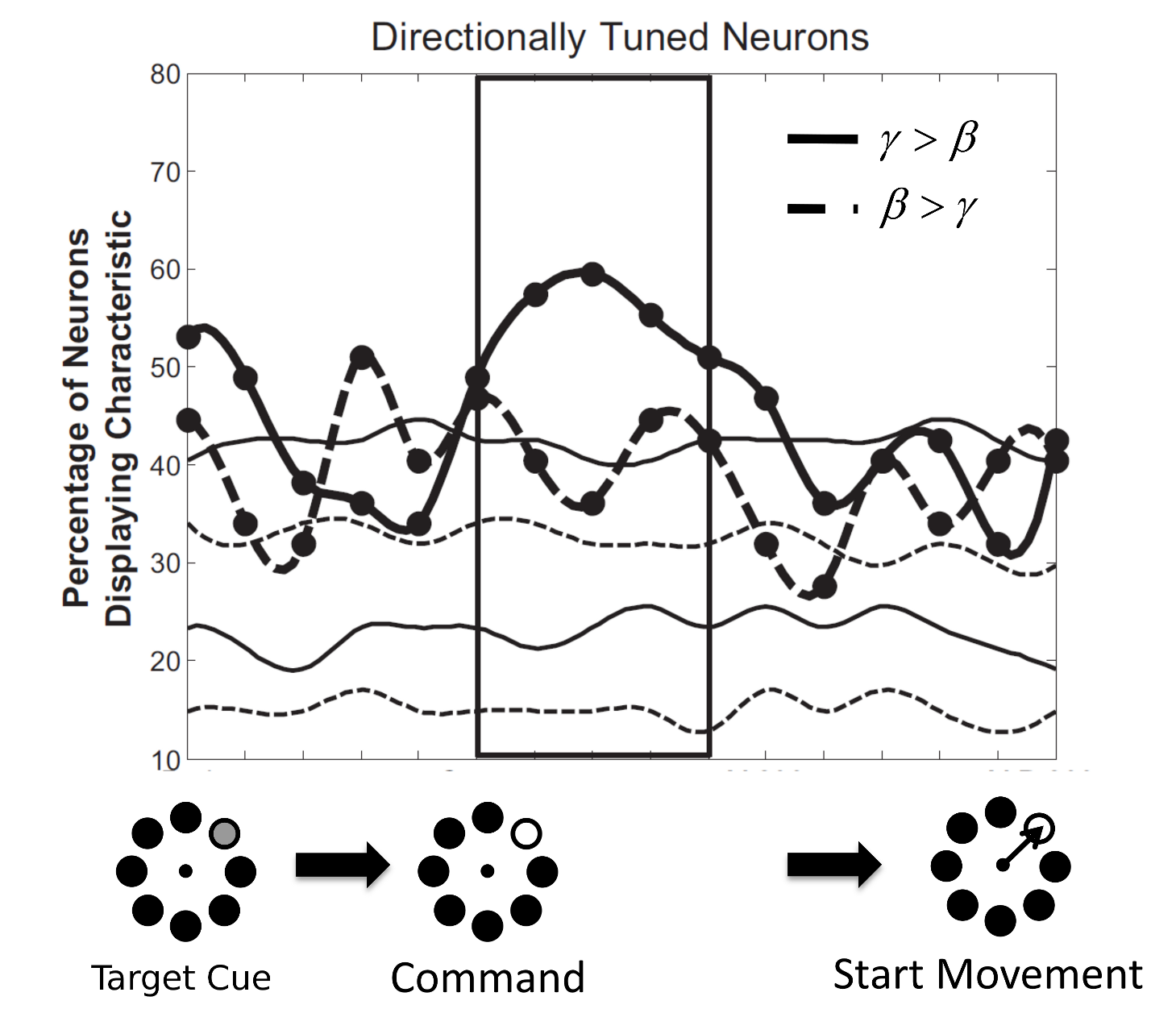

Supplement: Figure S4 — Neurons in GPi show a crossover effect during the planning phase. In this experiment, two primates executed a directed hand-movement task. From the figure, we can see that the percentage of neurons displaying more power in gamma band compared to beta band increases just after the final command is given. This may be because the GPi output is the modulating input to the relay neurons in motor thalamus, and a increase in the frequency of the modulating input may allow a certain motor plan to be relayed back to cortex and downstream to brain stem to ultimately get executed. This figure has been taken from [63]. The thin solid and dotted lines are the and confidence bounds obtained by randomization of the spike trains. (TIFF) [file pcbi.1002626.s004.tiff]
